# Supplementary material for: Tailoring and Long-Term Preservation of the Properties of PLA Composites with “Green” Plasticizers
Source: Polymers (Basel). 2022 Nov 10;14(22):4836. doi: 10.3390/polym14224836 (PMC9696962; doi:10.3390/polym14224836)
Supplement: Supplementary file 1 [file polymers-14-04836-s001.zip › polymers-1966953-supplementary.pdf]

Supplementary Material (online publication)

Tailoring and Long-Term Preservation of the Properties of PLA Composites with "Green" Plasticizers

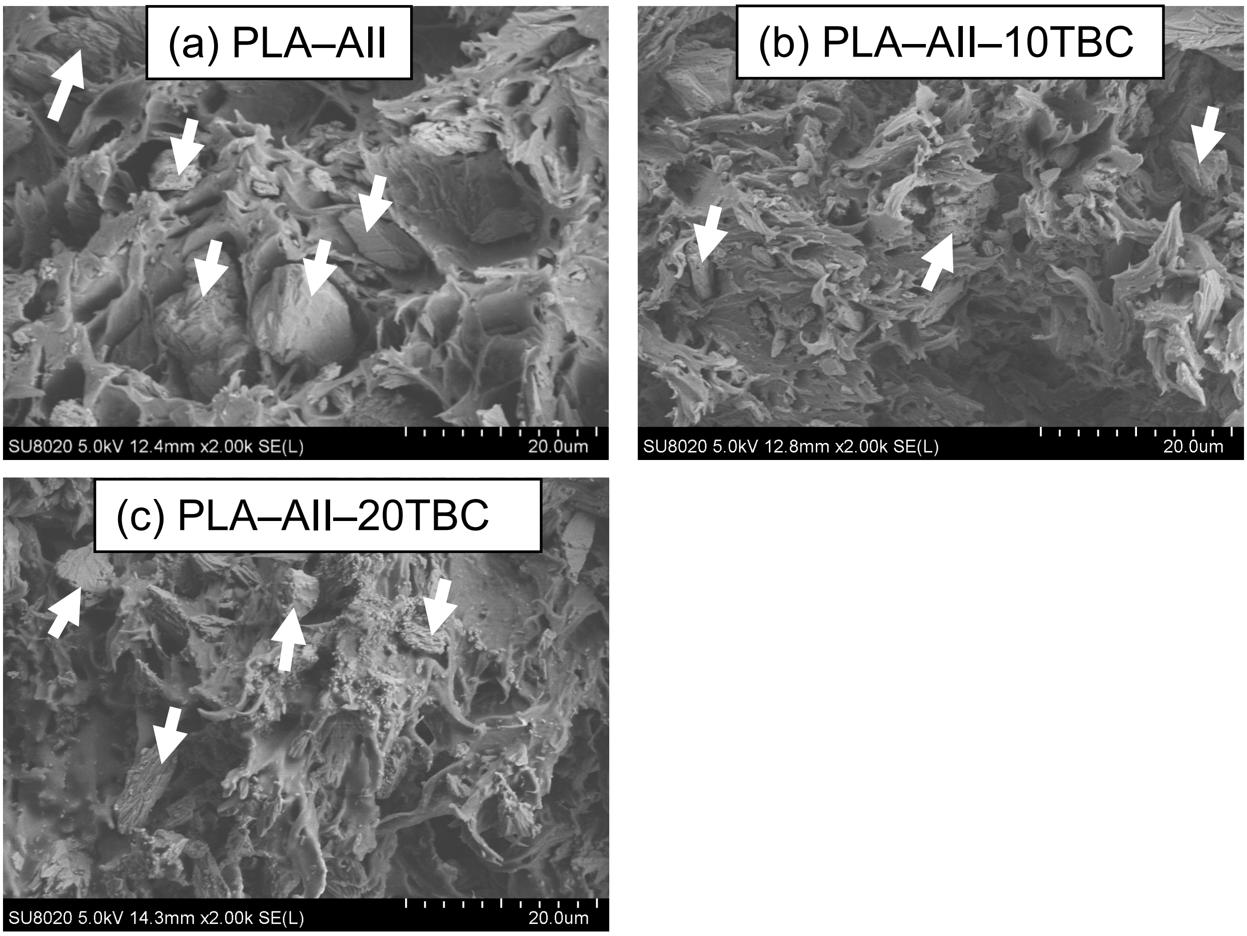

Figure S1. (a–c) SEM pictures (SE mode) on the surfaces of samples fractured during tensile testing: (**a**) unplasticized composites (PLA–AII) and plasticized composites with (**b**) 10% and (**c**) 20% TBC. *(For more easy interpretation, the presence of microparticles is evidenced by arrows.)*

Short comments: *The fractures are obtained at a tensile speed of 10 mm/min. Presence of more clear interfacial zones (PLA–filler) and a characteristic brittle behavior, without to have the evidence of plastic deformation is seen for the composites without plasticizer (PLA–AII) from Fig.S1a. By contrary, by plasticizing a more ductile fracture is observed (Fig. S1b and Fig. S1c), with specific features connected to the presence of elongated fibrils and stretched zones formed by the deformation of plasticized PLA matrix during mechanical solicitation. However, a slight better distribution of microparticles can be apparently ascribed to the plasticized compositions, whereas on the other hand,*

it is assumed that the tensile parameters are highly determined by the proprieties of plasticized matrix, with only some minor contribution from the part of the filler (NB: usually credited with a reinforcing role in PLA–AII composites).

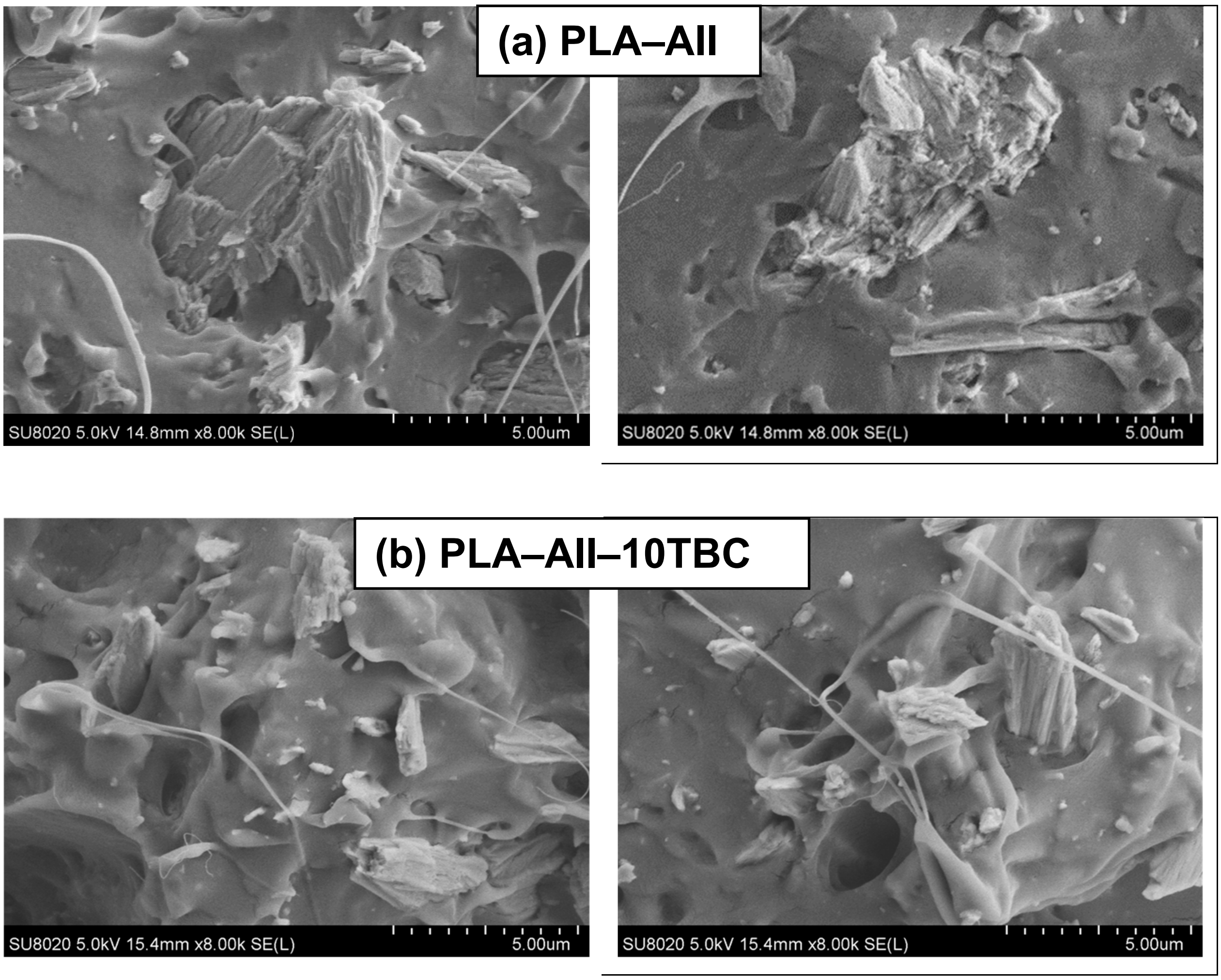

Figure S2. (**a,b**) SEM pictures (SE) on the surfaces of samples fractured during impact testing: (**a**) unplasticized composites; (**b**) plasticized composites with 10% TBC. *(NB: The plasticized composites with 15–20% TBC are not broken by impact testing at RT).*

Short comments: *First, it is noteworthy mentioning that during the tensile testing the composites have been stretched at low speed (10 mm/min) until they break, whereas in impact tests a sudden shock is given to the material at high energy/deformation rate (respectively, 3.9J and 3.46 m/s), and this leads to the fracture of more brittle composites. Once more, the presence of AII microparticles (of higher or lower dimension) distributed within the fragile PLA matrix is clearer visualized in the absence of plasticizer. Moreever, the SEM images (Fig. S2a) indicate the existence of distinct interfacial zones between components, i.e., regions of debonding or shear yielding, with a potential role in dissipating*

the energy of impact solicitation. By contrary, the higher plasticity/ductility of plasticized PLA matrix is evidenced by the presence of numerous elongated/stretched regions (Fig. S2b). Still, the comparative SEM pictures (Fig. S2a vs. Fig. 2b) suggest differences regarding the behavior of composites during impact solicitation. Here will not exclude for the discussion the hypothesis that by plasticizing the melt viscosity is significantly decreased, with potential effects in better filler dispersion and more intimate contact between components (PLA and AII). As in tensile tests, the mechanical properties of composites appear to a great extent to be determined by the high ductility/plasticity of the plasticized PLA matrix.

Supplementary Material: Figure S3 (a–d)

Morphology of composites produced using twin screw extruders (TSE).

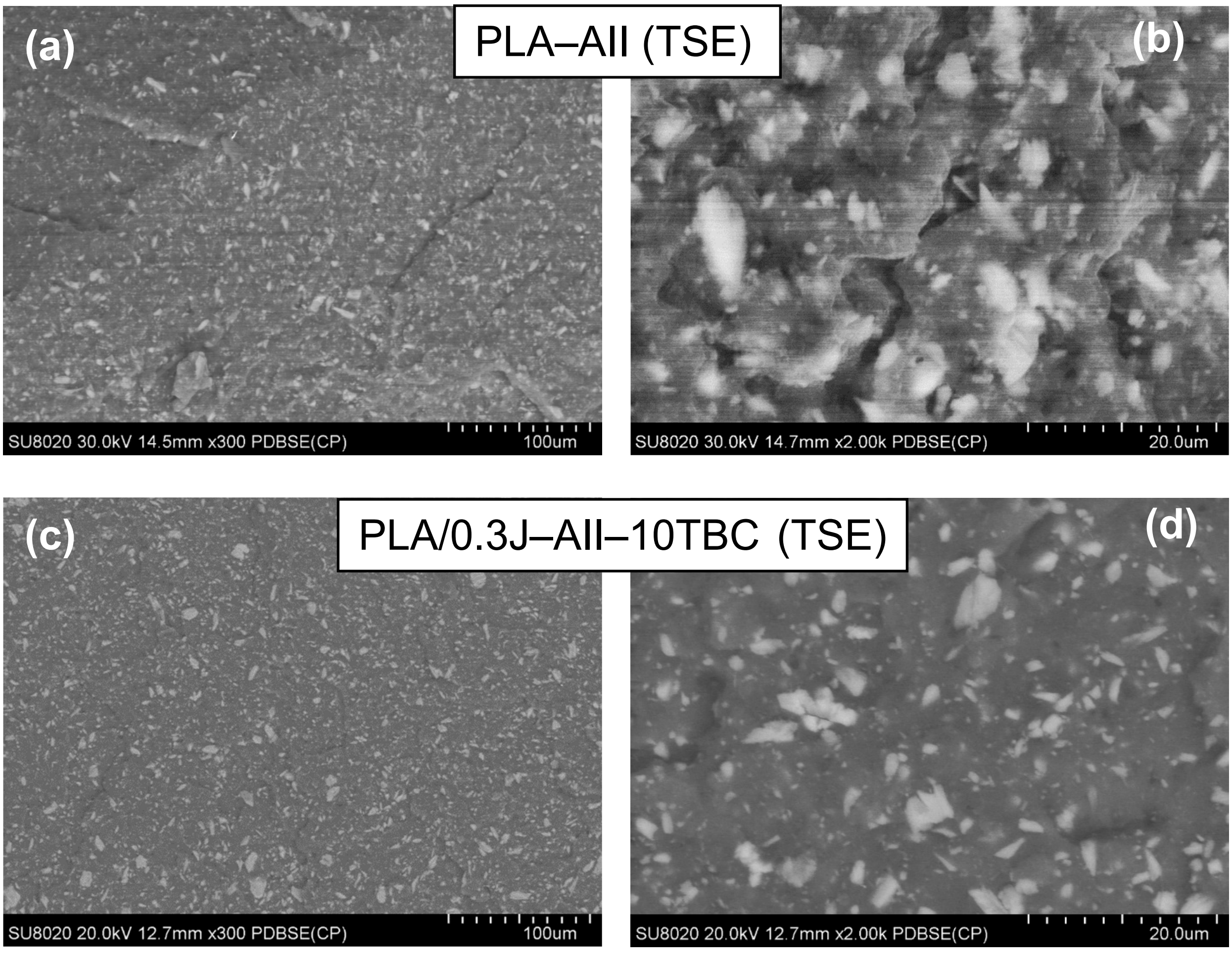

Figure S3. (**a–d**) SEM (BSE) at different magnifications on the cryofractured surfaces of composites produced using twin–screw extruders: (**a**,**b**) PLA–AII (TSE) and (**c**,**d**) PLA/0.3J–AII–10TBC (TSE).

Short comments: *Regarding the morphology of composites obtained by melt–compounding/REX using twin screw extruders, it is important to note that in both cases, the distribution of AII (30 wt.%) trough PLA matrix (in presence or not of plasticizer) is remaining adequate, without to evidence the presence of aggregates of microparticles (Fig. S3a vs S3c). Still, only minor differences regarding the better dispersion of microparticles in the plasticized composites are suggested by some SEM pictures performed at higher magnification (e.g., Fig. S3d).*

Supplementary Material: Figure S4

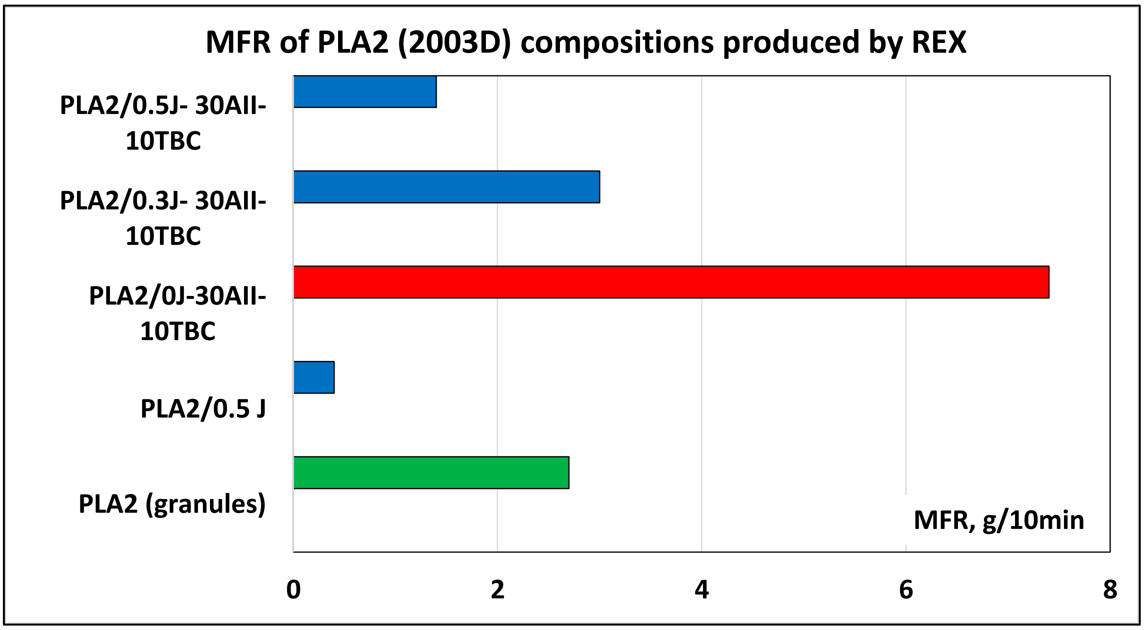

Figure S4. Melt flow rate values (2.16 kg, 2.1 mm, 190 °C) to illustrate the effectiveness of Joncryl addition by REX in selected PLA2 compositions.

Short comment: *PLA 2003D (NatureWorks LLC) abbreviated as PLA2, is a high molecular weight /high viscosity grade (MFR at 210 °C of 6 g/10min) used as second alternative (i.e., amorphous PLA matrix) and tested following the same experimental pathways shown in the study. By comparing to PLA 4032D, this grade (i.e., 2003D) has lower melting temperature (152-154 °C) and reduced speed of crystallization, typical properties ascribed to a PLA having higher D-enantiomer content (i.e., 4.2%), therefore by processing is leading to mostly "amorphous" products (films, tubes/straws, etc.). Moreover, PLA2 is specifically designed for use in food packaging and food service-ware applications, whereas the extruded films can be used to obtain different products by thermoforming.*

Summarizing, using PLA2 as matrix is once more proved the efficiency of Joncryl in tailoring the rheology/MFR of plasticized composites. Indeed, as it comes out from Fig. S4, the addition of 0.3–0.5% Joncryl by REX in plasticized compositions (PLA– 30% AII– 10% TBC) is counterbalancing the undesired effect of plasticizer in increasing the melt–fluidity (MFR is decreasing from about 7.5 to 1.5–3 g/10min). Nevertheless, the sample PLA2-/0.3J–30AII–10TBC was produced by REX in larger

quantities. Because of its higher melt strength/viscosity (MFR= 3 g/10 min), it can be successfully used to produce by extrusion tubes (straws), films (e.g., PLA mulch films) or for thermoforming.
